# Protective Effect of L-arginine Against Necrosis and Apoptosis Induced by Experimental Ischemic and Reperfusion in Rat Liver

**DOI:** 10.4103/1319-3767.45356

**Published:** 2009-07

**Authors:** Pronobesh Chattopadhyay, Gunjan Shukla, Arun Kumar Wahi

**Affiliations:** 1 Cellular and Microbiology Laboratory, College of Pharmacy, IFTM, Lodhipur Rajput, Moradabad - 244 001, UP, India; 2 Pharmacy Group, Birla Institute of Technology and Sciences, Pilani - 333 031, Rajasthan, India; 3 National Biotechnology Center, Indian Veterinary Research Institute. Izatnagar - 243 312, UP, India

**Keywords:** Apoptosis, *Bcl-2* gene, ischemic and reperfusion injury, L-arginine

## Abstract

**Background/Aim:**

To study the effect of L-arginine on apoptosis and necrosis induced by 1-h ischemia followed by 3-h reperfusion.

**Materials and Methods:**

Adult Wistar rats underwent 60 min of partial liver ischemia followed by 3-h reperfusion. Eighteen Wistar rats were divided into sham-operated control group (I) (*n* = 6), ischemia and reperfusion (I/R) group (0.9% saline (5 mL/kg, orally) for 7 days) (II) (*n* = 6), and L-arginine-treated group (10 mg/kg body weight daily orally for 7 days before inducing ischemia-reperfusion maneuver) (III) (*n* = 6). Apoptotic and necrotic hepatocytes, nitric oxide levels in hepatocytes, Bcl-2 mRNA, and Bcl-2 protein were measured. Liver injury was assessed by plasma alanine transaminases (ALT), aspartate transaminases (AST), liver histopathology, and electron microscopy.

**Results:**

An ischemic and reperfusion hepatocellular injury occurred as was indicated by increased serum ALT, AST, histopathology, and electron microscopy. Apoptosis and necrosis associated marker gene Bcl-2 mRNA and protein expression were decreased in I/R group. Pretreatment with L-arginine significantly decreased serum ALT and AST level and apoptotic and necrotic cells after 1 h ischemia followed by 3 h of reperfusion. Nitric oxide production in hepatocytes was increased twofold by L-arginine treatment when compared with I/R group. Histopathology and transmission electron microscopy (TEM) studies showed markedly diminished hepatocellular injury in L-arginine-pretreated rats during the hepatic I/R.

**Conclusion:**

Thus, it may be concluded that L-arginine afforded significant protection from necrosis and apoptosis in I/R injury by upregulated Bcl-2 gene and nitric oxide production.

Ischemic reperfusion (I/R) injury is a phenomenon whereby cellular damage occurs because of oxygen delivery into the liver tissue. This form of injury in the liver was recognized as a clinically important pathological disorder.[1] Deprivation of oxygen, nutrients, or growth factor may be important for the cause of I/R injury resulting in cell atrophy and apoptosis. The acute injury phase (early phase), which is characterized by liver injury occurring within 1-6 h after reperfusion, is associated with Kupffer cell activation, release of the proinflammatory cytokines, and the generation of reactive oxygen species (ROS).[1] Apoptosis and cell atrophy have been indicated as an important mode of cell death during hepatic I/R injury.[2] Previous study showed that the initial peak of apoptosis and necrotic cells was after 1 h ischemia, followed by 30 min reperfusion, and the second peak was after 1 h ischemia and 3 h reperfusion, followed by gradual decline of necrosis and apoptosis.[3] Bcl-2 family of gene plays a major role in determining the ultimate sensitivity or resistance of cells to myriad stimulus and insults that induce apoptosis in mitochondria.[4] Oxidant stress-induced cell killing involves the oxidation of pyridine nucleotides, accumulation of calcium in mitochondria, and superoxide formation by mitochondria, which ultimately lead to the formation of membrane permeability transition pores and breakdown of the mitochondrial membrane potential.[5]

Arginine is an α-amino acid. The L-form is one of the 20 most common natural amino acids. In mammals, arginine is classified as a semiessential or conditionally essential amino acid, depending on the developmental stage and health status of the individual. Nitric oxide (NO) is a potent vasodilator synthesized from L-arginine and diffuses freely across cell membranes and acts intracellularly by the activation of guanylate cyclase. NO is an inducer of vasodilatation at the level of the sinusoid as well as at presinusoidal sites.[6] In addition to its vasodilatory effect, NO reacts with superoxide to form the potent oxidant peroxynitrite.[7] NO inducer of vasodilatation at the site of sinusoid as well as at presinusoidal sites,[8,9] which causes vasodilatation in Kupffer cells. NO also reacts with superoxide to form the potent oxidant peroxynitrite.[10] Inducible nitric oxide synthase (iNOS) from hepatocytes activates Kupffer cells to produce nitric oxide (NO). We hypothesized that the modulation of vasodilatation by NO could explain the protective effects of liver from I/R induced injury.

The effect of L-arginine therapy on hepatic ischemia has not yet been completely elucidated. Therefore, it was decided to investigate the role of nitric oxide donor L-arginine in I/R-induced injury, and the present study was designed to ascertain whether differences in the production of NO by hepatocytes cells could explain the differences in necrosis and apoptosis found in I/R injury. Further pathological changes in the different experimental and sham-operated control groups were correlated with histopathology and electron microscopy studies.

## MATERIALS AND METHODS

Collagenase, L-arginine, and bovine serum albumin (BSA) were procured from Hymedia (Mumbai, India) and Trizol from GIBCO (Germany). Primers were procured from Integrated DNA technologies (Inc.milpitas, CA). Ampli Taq polymerase dNTP, Ampli Taq polymerase, and agarose gels were procured from Amersham Pharmacia Biotech (Uppsala, Sweden). Peroxide-labeled goat anti-rabbit secondary antibodies were procured from Biosource international (New York, USA), and alanine aminotransferase (ALT) and aspartate aminotransferase (AST) were procured from Merck India Ltd (India). All other chemicals were obtained from Sigma (St. Louis, MO).

### Animals

All animals received care in compliance with the Guide for the Care and Use of Laboratory Animals (National Institutes of Health Publication No. 85-23, revised 1985). Experimental protocols were reviewed and approved by Institutional Use and Care Committee. Adult Wistar rats (Indian Veterinary Research Institute) weighing between 250 and 280 g were fed a standard diet and acclimatized in the animal housing area for 1 week before experimentation.

### Induction of ischemic and reperfusion injury

The hepatic I/R protocols were performed as described in a previous study by Chattopadhyay *et al*.[11] After the induction of anesthesia (urethane 10 mg/kg i.p.), the liver of each rat was exposed through a midline laparotomy. Complete ischemia of the median and left hepatic lobes was produced by clamping the left branches of the portal vein and the hepatic artery for 60 min. The right hepatic lobe was perfused to prevent intestinal congestion. After the period of ischemia, the ligatures around the left branches of the portal vein and hepatic artery were removed. To accurately evaluate the blood flow of the median and left hepatic lobes after ischemia, the right branches of the portal vein and the hepatic artery were ligated to prevent shunting to the right lobe after reperfusion and perfused for 3 h. The wound was closed with 3-0 silk suture. Sham-operated animals were similarly prepared except that no ligature was placed to obstruct the blood flow to the left and median hepatic lobes. Instead, the blood flow to the right lobe of the liver was occluded. In all groups rats were sacrificed after 1-h ischemia followed by 3-h reperfusion. A total of 18 Wistar rats were equally divided into three groups (n=6 each group). Group I (sham-operated control group) and Group II (ischemia and reperfusion group) were given 0.9% saline (5 mL/kg, orally) for 7 days. Group III was pre-cotreated with L-arginine (100 mg/kg orally) for 7 days before induced ischemia-reperfusion maneuver.

### Peripheral blood and tissue procurement

Blood samples were obtained from the right ventricle via left anterior thoracotomy at the time of sacrifice. Blood was collected in a sterile syringe without anticoagulant and centrifuged at 2000 g to separate the serum. The serum samples were stored at −20°C until use for AST and ALT assays. A portion of the ischemic and nonischemic liver lobe was fixed in buffered 10% formalin and Karnovsky's solution for histopathology and TEM studies, respectively. Another portion of ischemic and nonischemic liver lobe were snap-frozen in liquid nitrogen and stored at −70°C for reverse-transcriptase polymerase chain reaction (RT-PCR), fluorescence microscopy, and flow cytometry.

### Biochemical analysis

#### Isolation of hepatocytes

Viable hepatocytes were prepared by the collagenase perfusion modified method Kreamer.[12] Briefly, after killing the rats, the left lobes were cannulated and perfused at 6 to 8 mL/min with Earle balanced salt solution (EBSS) containing 0.25 mM EDTA (Na)_2_ for 4 min. The EDTA (Na)_2_ was flushed out with EBSS for 4 min before perfusion with EBSS containing 1 mM calcium chloride, 0.68 mg/mL collagenase H, and 0.07 mg/mL trypsin inhibitor for 8–12 min until the cell matrix was seen to break. The cells were gently teased out of the liver capsule into Williams medium E (WME), pH 7.4, containing 1% (wt/vol) BSA, and the cell suspension was filtered through a nylon mesh and centrifuged for 3 min at 100 g. The supernatant was discarded, the cell pellet was resuspended in WME containing 1% BSA (w/v), and the centrifugation step was repeated twice. The final cell pellet was resuspended in WME containing 0.1% BSA, and the viability was obtained using the trypan blue exclusion method.

### Measurement of NO and liver function enzymes

NO was measured as described by Hibbs *et al.*[13] Serum was used for the assay of alanine aminotransferase (ALT) [EC 2.6.1.2] and aspartate aminotransferase (AST) [EC 2.6.1.1] measured by using estimated by Merck kits (Merck India Ltd, Mumbai, India) according to the manufacture's instructions.

### Reverse-transcriptase polymerase chain reaction

Total cytoplasmic RNA of each group was isolated from liver tissue by the Trizol method (according to GIBCO specification). RNA quality was determined by spectrophotometric analysis (OD 260/280) and quantified at 260 nm. The experiment was performed using a light cycle rapid thermal cycle (Epindrop, Germany). The RT reaction was amplified using Taq DNA polymerase and primers to rat Bcl-2 C DNA (sense: 5'-CGT-CAT-AAC-TAA-AGA-CAC-CCC-3'; antisense: 5'-TTC-ATC-TCC-AGT-ATC-CGA-CTC-3'). The PCR profile was set at 94°C melting, 55°C annealing, and 72°C extension for 1 min, and semi- quantization was optimized to 35 cycles. GAPDH (glyceraldehyde 3-phosphate dehydrogenase) transcript abundance was used as an endogenous control, and Bcl-2 transcript abundance was normalized.

### Western blot analysis

Cell lysates were prepared from liver and lysed in a buffer contains 1% triton X-100 10 mM Tris (PH 7.4), 150 mM NaCl, 2 mg/mL aprotinin, and 10 mM phenylmethyl sulfonyl fluoride (PMSF). Protein samples (50 *μ*g) were analyzed by sodium dodecyl sulfate (SDS) polyacrylamide gel electrophoresis (PAGE) under reducing condition transferred overnight to a nylon membrane. Both were incubated with rabbit anti-mouse/rat Bcl-2 primary antibody, followed by peroxides-labeled goat anti-rabbit secondary antibody (Bio source international, USA), and bound antibody were detected by enhanced chemiluminescences. Bands were quantitatively measured by densitometry analysis system.

### Morphological evaluation light microscope assay

Serial slices of liver tissues were prepared from rat in each group and stained with hematoxyline-eosin (HE) and then observed under light microscope at ×200 or ×400 magnifications.

### Transmission electron microscopy assay

Liver tissues were fixed in Karnovsky's solution pH 7.4 for 4 h at 4°C. After fixation and an overnight wash in sodium cacodylate buffer at 4°C, the specimens were postfixed with 1% osmium tetroxide in 0.1 M phosphate buffer (pH 7.4), dehydrated in ethanol and then embedded in Araldite resin, and semithin sections (1 *μ*m) were removed for optical microscopy. Ultrathin sections (40-60 nm thick) were placed on copper mesh grids (200 mesh) and doubly stained with uranyl acetate and lead citrate. Sections were examined using a transmission electron microscope (Moragagni 268D by Netherlands), and photomicrographs were taken.

### Fluorescence microscopy

Cell morphology of IR-induced apoptosis was investigated by staining the cells with a combination of fluorescent DNA binding dyes Annexin-V and ethidium bromide (EB). Briefly, cells were harvested and washed with PBS after the isolation of hepatocytes from different group. After staining with 100 *μ*g/ml AO/EB for 5 min, the cells were observed under a fluorescence microscope (Olympus).

### Flow cytometry analysis

Hepatocytes(1×10^9^/L) then washed with PBS, exposed to propinum iodide (PI) 50 mg/L, 0.1% Triton X-100, 0.01 mmol/L EDTA (Na)_2_ and RNase 50 mg/L at normal temperature in darkness for 12–24 h. Specimen were then presented to the FACS-420 Flow Cytometry Analyzer (Becton Dickinson and Company, New York, USA) to evaluate apoptosis levels. The apoptotic and necrotic cells were finally analyzed with the Modfit 3.0 DNA software on the basis of percentage of hepatocytes staining with PI and cytogram (distribution).

### Statistical analysis

All values were expressed as mean ± SD. Differences in mean values were compared using SPSS 11.0 by one-way ANOVA and Student-Newman-Keul (SNK) test. *P* < 0.05 was considered as statistically significant.

## RESULTS

### Effect of L-arginine in levels of ALT and AST

The ALT and AST levels in the sham-operated control rats were 84.72 ± 8.20 and 56.02. ± 9.52, respectively, which increased to 1507.36 ± 30.58 and 817.40 ± 14.52, respectively, after 1-h ischemia followed by 3-h reperfusion. The significant increase in ALT and AST activities that occurred after 1-h ischemia followed by 3-h reperfusion in the I/R group was significantly suppressed by the administration of 100 mg/kg L-arginine [Table 1].

**Table 1:** Effect of L-arginine in activities of ALT and AST in the liver of sham and experimental groups of rat

Groups	ALT#	AST#
Sham (group I)	84.72 ± 8.20	56.02 ± 9.52
I/R injury (group II)	1507.36 ± 30.58**	817.40 ± 14.52**
L-arginine + I/R injury (group III)	187.21 ± 14.52*##	105.10 ± 8.92**##

Results are expressed as mean ± SD (n = 6); Significantly different

* (*P* < 0.05,

** *P* < 0.01) from sham-operated group. Significantly different

# (*P* < 0.05,

## *P* < 0.01) from I/R injury group.;

# Expressed in IU/L

### Effect of L-arginine on the production of NO, necrosis, and apoptosis

In the sham-operated control rats, the NO level remained constant at 11.09 ± 4.8 pmol NO formed/min/mg of protein throughout the experiment. In the I/R rats, NO level decreased to 8.20 ± 1.5 pmol/min/mg of protein after 1-h ischemia followed by 3-h reperfusion. This reduction in NO was attenuated by 100 mg/kg L-arginine [Table 2].

**Table 2:** Effect of L-arginine on the production of NO, apoptotic and necrotic cell death rates in liver after 1-h ischemia and 3-h reperfusion (flow cytometry analysis)

Group	‡No production	#Necrotic cells	#Apoptotic cells
Sham-operated control (group I)	11.09 ± 4.81	1.02 ± 0.32	0.70 ± 0.08
Ischemia and reperfusion (group II)	8.20 ± 0.57**	21.54 ± 7.18*	26.44 ± 6.02*
L-arginine + I/R injury (group III)	26.57 ± 6.30*#	5.68 ± 0.86**##	9.70 ± 0.93 **##

Results are expressed as mean ± SD (n = 6); Significantly different

* (*P* < 0.05,

** *P* < 0.01) from sham operated group; Significantly different

# (*P* < 0.05,

## (*P* < 0.01) from I/R injury group;

‡ Expressed in pmol/min/mg of protein;

# Expressed in % of normal hepatocytes

**Figure 1:**
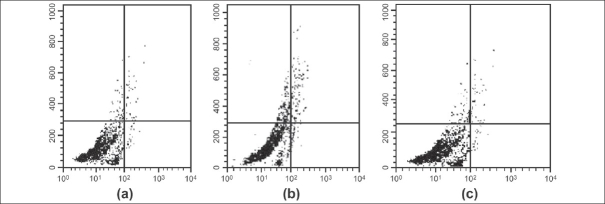
I/R-induced apoptosis in hepatocytes. Flow cytometry analysis assessing apoptosis after PI staining. (a) Flow cytogram of sham operated control rats (I). (b) Flow cytogram of ischemic and reperfused rats (II). (c) Flow cytogram of L-arginine-treated rats (III)

The percentage of necrotic and apoptotic cells were 1.02 ± 0.32 and 0.70 ± 0.08, respectively [Figure 1a]. In the I/R rats, necrotic and apoptotic cells were increased to 21.54 ± 7.1 and 26.44 ± 6.0, respectively [Figure 1b]. The decreases were restored to the level observed in sham-operated control rats by 100 mg/kg L-arginine [Figure 1c, Table 2].

### Effects of L-arginine in expression of Bcl-2 mRNA and protein

**Figure 2:**
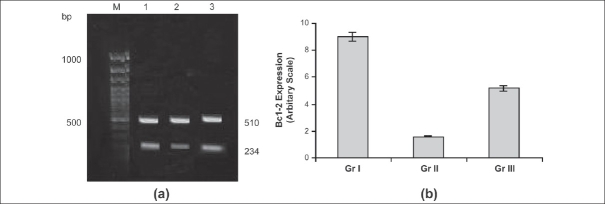
Bcl-2 gene expression. (a) Representative photograph of the expression of Bcl-2 gene by RTPCR analysis. Bcl-2 gene expressed at 234 bp and house keeping gene glyceraldehydes-3 phosphate dehydrogenase expressed at 510 bp. Lane M: Marker, Lane 1: Sham-operated control group (I), Lane 2: I/R rat (II), Lane 3: L-arginine treated (III); (b) The expression of Bcl-2 gene after 1 h ischemia and 3 h reperfusion. Data are expressed as the mean ± SD

**Figure 3:**
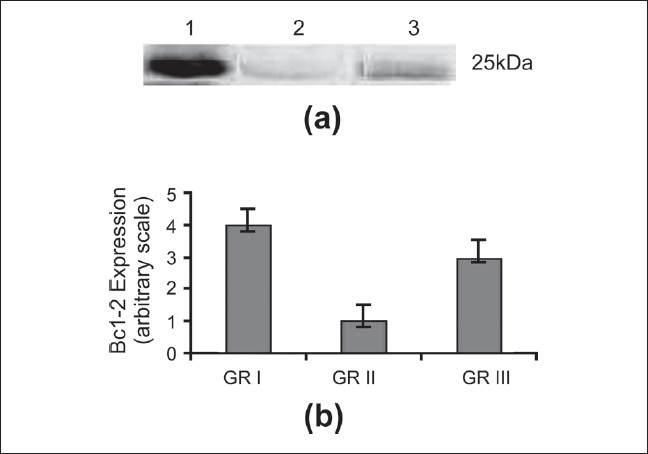
The expression of Bcl-2 protein. (a) Representative photograph of the expression of Bcl-2 protein using western blot analysis. Lane M: Marker, Lane 1: Sham-operated control group (I), Bcl-2 was strongly expressed in the sham-operated liver tissues. Expression level decreased after 1 h ischemia followed by 3 h reperfusion in group II. Lane3: L-arginine treated (III); (b) The expression of Bcl-2 protein after 1 h ischemia and 3 h reperfusion. Data are expressed as the mean ± S.D

To investigate the mechanism of L-arginine on necrosis and apoptosis, mRNA and protein expression of the Bcl-2 in ischemic liver tissue followed by 3 h reperfusion was measured by RT-PCR and Western blot techniques. Appreciable levels of Bcl-2 mRNA and protein expression were observed in liver tissues of L-arginine treated with the matching sham-operated rat. Ischemia for 1 h followed by 3 h reperfusion resulted in significant (*P* < 0.05) decreases in mRNA and protein expression of Bcl-2 when compared with L-arginine-treated rats [Figures 2 and 3].

### Effects of L-arginine in histopathological changes of liver tissue

**Figure 4:**
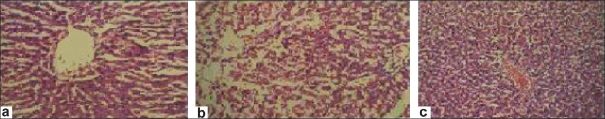
Showing histopathology of liver stained with hematoxylin-eosin. (a) Sham-operated control group (I) (H and E, ×400); (b) I/R-injured rats (II) (H and E, ×400); (c) L-arginine-treated group (III) (H and E, ×400)

No morphological damage was observed in any of the rats in sham-operated control group [Figure 4a]. In I/R-treated rats, the hepatocytes were markedly swollen, with striking vacuolization. In addition, areas of necrosis and cell infiltration were noted [Figure 4b]. L-arginine treated rats showed well-preserved liver parenchyma with hepatocytes extending from the central vein. Also arginine-treated rats showed the regular sinusoidal structures and normal morphology, without any signs of congestion [Figure 4c].

### Effects of L-arginine in ultrastructural changes of hepatocytes

**Figure 5:**
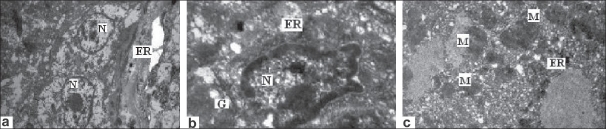
Transmission electron microscope analysis liver sections following I/R. (a) Sham-operated control rat (I); (b) I/R-induced rat (II); (c) L-arginine-treated group (III). Endoplasmic reticulum (ER); mitochondria (M); glycogen (G); nucleus (N) (uranyl acetate and lead citrate stain, ×8000)

After 1-h ischemia followed by 3-h reperfusion, mitochondria in the hepatocytes were severely swollen, and the number of cristae was reduced. Also, after I-h ischemia followed by 3-h reperfusion, the number of smooth endoplasmic reticulum and secondary lysosomes was increased, nucleus was not well marked, and glycogen granules decreased [Figure 5b]. Degenerative changes of hepatocytes were attenuated by L-arginine [Figure 5c], which was comparable to sham-operated control rats [Figure 5a].

### Effect of L-arginine in apoptosis (fluorescence microscopy)

**Figure 6:**
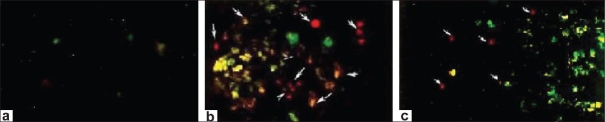
Fluorescence microscopy of hepatocytes stained with ethidium bromide and Annexin-V (100 mg/mL). Viable cells observed with green nuclear fluorescence and apoptotic cells containing an orange nucleus (marked arrow) and exhibiting chromatin condensation (magnification, ×200). (a) Sham-operated group (I); (b) Ischemia followed by reperfusion group (II); (c) L-arginine-treated group (III)

Apoptosis was evaluated based on some distinct morphological features such as cell shrinkage, chromatin condensation, oligonucleosomal DNA fragmentation, and finally the breakdown of the cell into smaller units (apoptotic bodies). Reperfusion of the ischemic liver caused severe hepatocellular apoptosis after 1-h ischemia followed by 3-h reperfusion in the I/R group [Figure 6b]. In contrast, in sham-operated rats, there was no sign of apoptosis [Figure 6a]. The increase in apoptosis that occurred after 1-h ischemia followed by 3-h reperfusion in the I/R group rats was significantly suppressed by the administration of L-arginine [Figure 6c].

## DISCUSSION

The results indicate that after 1 h ischemia followed by 3 h reperfusion, the liver function was improved by L-arginine pretreatment and could be protected against liver damage from necrosis and apoptosis induced by I/R injury. In addition, the present data indicate an association between expression of Bcl-2 and hepatocellular necrosis and apoptosis during L-arginine treatment. Hepatocytes NO production was increased significantly in L-arginine- treated group when compared with sham-operated rats. Nitric oxide, known as an endothelium-derived relaxing factor, is formed from the terminal guanidino nitrogen atom of L-arginine.[14,15]

In this study, we found that suppression of Bcl-2 showed increased number of necrotic cells ad apoptosis. These data are consistent with a previous study finding that overexpression of Bcl-2 reduced ischemia/reperfusion injury.[16] In addition, this study provides a new insight into the mechanism by which L-arginine mediates hepatoprotective. The family of Bcl-2-related proteins plays a key role in the regulation of apoptosis. Bcl-2, a member of the Bcl-2-related gene, can promote cell survival through protein-protein interactions with other Bcl-2-related protein family members. Recent studies indicate that overexpression of Bcl-2 gene could reduce heaptocellular apoptosis after reperfusion and protect against hepatic I/R injury.[17] Bcl-2 homodimers inhibit cell death. Therefore, increase in the expression of Bcl-2 may determine survival following apoptotic stimuli and attenuate the antiapoptotic effect of Bcl-2 gene by reducing postischemic apoptosis.[16] However, attempts to protect against hepatic I/R injury by alleviating the possible detrimental effects of portal venous congestion have not achieved satisfactory results. Previous study showed that apoptosis caused by I/R injury may be due to endonuclease activity or by acting on cell organelles, alternating signal transduction pathways or affecting the intracellular enzymes responsible for proper functioning and survival of the cell.[18] Also, it was demonstrated in our previous study that DNA content and decrease in cytochrome P 450 in ischemia and reperfusion injury presumably results in an increased activity of TNF-α by the toxic effect of ischemia and reperfusion. TNF-α may mediate direct toxicity to mitochondria and induce apoptosis or cell death.[1] The ultrastructural changes observed microscopically in I/R-injured rats showed severe degeneration of cellular architecture. Pretreatment with L-arginine showed considerable prevention in the ultrastructural alternations including the distribution of mitochondrial fine structure.

Histological examination of the liver revealed regular sinusoidal structures in group III, as opposed to swollen cells with marked vacuolization seen in group II as a result of stimulating action of NO production by L-arginine. The results showed that treatment with L-arginine improved the degree of the heaptocellular structure.

Nitric oxide, known as an endothelium-derived relaxing factor, is formed from the terminal guanidino nitrogen atom of L-arginine by NO synthase.[14] Nitric oxide binds to the heme moiety of guanylate cyclase and increases its activity by 400-fold by catalyzing the conversion of guanosine triphosphate to cyclic guanosine monophosphate (cGMP). Elevation of cGMP relaxes the smooth muscles in blood vessels including the genitourinary tract, inhibits platelet aggregation and adhesion, and blocks the adhesion of white cells to the blood vessel wall.[19] The results of the present study suggest that the treatment of L-arginine might have a possible role at a subcellular level in preventing I/R-induced necrosis and apoptosis.

In conclusion, the enhanced production of NO through the administration of L-arginine reduces necrosis and apoptosis by attenuated Bcl-2 expression in I/R-induced injury. This study also shows that L-arginine protects hepatobiliary function and the ultrastructure of liver in hepatic I/R-induced injury. Further studies are in progress to elucidate the molecular mechanisms of L-arginine and their potential in the treatment and prevention of hepatotoxicity.
